# Interactions between nocturnal melatonin secretion, metabolism, and sleeping behavior in adolescents with obesity

**DOI:** 10.1038/s41366-022-01077-4

**Published:** 2022-02-09

**Authors:** Johanna Overberg, Laura Kalveram, Theresa Keller, Heiko Krude, Peter Kühnen, Susanna Wiegand

**Affiliations:** 1 Department of Pediatric Gastroenterology, Nephrology and Metabolic Diseases, Charité Universitätsmedizin Berlin, Berlin, Corporate Member of Freie Universität Berlin, Humboldt-Universität zu Berlin, and Berlin Institute of Health, Augustenburger Platz 1, 13353 Berlin, Germany; 2 Center for Chronically Sick Children, Charité - Universitätsmedizin Berlin, Corporate Member of Freie Universität Berlin, Humboldt-Universität zu Berlin, and Berlin Institute of Health, Augustenburger Platz 1, 13353 Berlin, Germany; 3 Institute of Biometry and Clinical Epidemiology, Charité - Universitätsmedizin Berlin, Corporate Member of Freie Universität Berlin, Humboldt-Universität zu Berlin, and Berlin Institute of Health, Reinhardtstraße 58, 10117 Berlin, Germany; 4 Institute for Experimental Pediatric Endocrinology, Charité Universitätsmedizin Berlin, Corporate Member of Freie Universität Berlin, Humboldt-Universität zu Berlin, and Berlin Institute of Health, Augustenburger Platz 1, 13353 Berlin, Germany

**Keywords:** Risk factors, Metabolic syndrome, Obesity

## Abstract

**Background/objectives:**

Sleeping behavior and individual prospensity in sleep timing during a 24 h period, known as chronotypes, are underestimated factors, which may favor the development of obesity and metabolic diseases. Furthermore, melatonin is known to play an important role in circadian rhythm, but was also suggested to directly influence metabolism and bodyweight regulation. Since disturbed and shifted sleep rhythms have been observed in adolescents with obesity, this study aimed to investigate potential interactions between melatonin secretion, chronobiology, and metabolism. In addition, the influence of artificial light especially emitted by electronic devices on these parameters was of further interest.

**Subjects/methods:**

We performed a cross-sectional study including 149 adolescents (mean age 14.7 ± 2.1 years) with obesity. Metabolic blood parameters (e.g., cholesterol, triglycerides, uric acid, and insulin) were obtained from patients and correlated with nocturnal melatonin secretion. Melatonin secretion was determined by measuring 6-sulfatoxymelatonin (MT6s), the major metabolite of melatonin in the first-morning urine, and normalized to urinary creatinine levels to account for the urinary concentration. Chronobiologic parameters were further assessed using the *Munich ChronoType Questionnaire*.

**Results:**

Subjects with insulin resistance (*n* = 101) showed significantly lower nocturnal melatonin levels compared to those with unimpaired insulin secretion (*p* = 0.006). Furthermore, triglyceride (*p* = 0.012) and elevated uric acid levels (*p* = 0.029) showed significant associations with melatonin secretion. Patients with late chronotype showed a higher incidence of insulin resistance (*p* = 0.018). Moreover, late chronotype and social jetlag were associated with the time and duration of media consumption.

**Conclusion:**

We identified an association of impaired energy metabolism and lower nocturnal melatonin secretion in addition to late chronotype and increased social jetlag (misalignment of biological and social clocks) in adolescents with obesity. This might point towards a crucial role of chronotype and melatonin secretion as risk factors for the development of pediatric and adolescent obesity.

## Introduction

Obesity has a multifactorial etiology and is affected by genetic background, lifestyle, and environmental factors. Besides reduced physical activity and high amounts of energy-dense food, insufficient sleeping behavior has been identified to favor the development of obesity in adults and adolescents [1, 2].

The pineal hormone melatonin plays a crucial role in the regulation of the biological clock and therefore in the sleep-wake rhythm. Its secretion is controlled by the master circadian clock located in the suprachiasmatic nucleus of the hypothalamus and follows a diurnal pattern with its main secretion during darkness [3]. While the highest concentrations of melatonin are observed during childhood, the secretion decreases during puberty and further with increasing age [4]. A failure of the pineal gland to grow and a tendency to calcify have been discussed as factors leading to a downregulation of pineal activity and to a loss of circadian rhythm with age [5, 6]. The modified secretion of melatonin during adolescence plays among other hormonal, genetic, and environmental (e.g., light exposure) factors an important role in regulating the individual timing of sleeping behavior, known as chronotypes [7]. During pubertal development, the sleep-wake behavior is physiologically shifted towards later chronotypes, which can lead to a misalignment of biological and social clocks in this age cohort [8, 9]. This phenomenon has been termed social jetlag [7, 10] and has been associated with increased risk for the development of obesity, metabolic disorders, and impaired mental health [11, 12].

One molecular link between sleep deficiency and metabolic diseases could be melatonin, which seems to have specific effects on metabolism and bodyweight regulation [13]. Further evidence regarding interaction between melatonin and glucose metabolism derives from genome-wide association studies: polymorphisms in the melatonin receptor 1B gene (*MTNR1B*) are associated with glucose intolerance, reduced β-cell function, and a higher risk for diabetes mellitus type 2 [14]. Additionally, McMullan et al. observed that nocturnal melatonin secretion was independently and inversely associated with insulin levels in a large women cohort without diabetes mellitus type 2, hypertension, or malignancy [15]. The same authors identified lower nocturnal melatonin secretion as an independent risk factor for the development of diabetes mellitus type 2 [16]. Furthermore, there is growing evidence that melatonin may significantly lower arterial systolic blood pressure and improve the lipid profile (decrease of LDL cholesterol and increase of HDL cholesterol) of animals and humans [17, 18].

The influence of media consumption on chronotype, melatonin secretion, and metabolism is of further interest. Studies showed that optical radiation at short wavelengths, which is emitted by devices, such as tablets, computers, or smartphones suppresses melatonin secretion, especially if used in the evening hours, leading to an altered sleeping behavior [19, 20]. This again may result in impaired physical and psychological health [20].

Most of the existing studies on that matter, only focus on partial aspects, are based on animal models, or were mainly conducted in adult cohorts. Especially, studies in children and adolescents are rare and inconclusive [21–24].

Therefore, the aim of the present study was to examine the interaction of chronobiologic parameters, melatonin secretion, and metabolism in a well-characterized adolescent cohort with obesity. We further wanted to assess the influence of time and duration of media consumption on these parameters.

## Subjects and methods

### Subjects

In this cross-sectional study, *n* = 149 adolescents with obesity between 10 and 17 years were recruited from the pediatric obesity outpatient clinic of Charité Universitätsmedizin Berlin (Germany) between August and December 2014. Inclusion criteria consisted of obesity (Body Mass Index (BMI) > 97th percentile) and age 10–17 years. Exclusion criteria included intake of melatonin or medication that is known to affect melatonin secretion (beta-blocker, antidepressants, NSAIDs, diuretics) or weight (e.g., corticosteroids) and concurring diseases affecting weight (Cushing´s syndrome, hypo-/hyperthyroidism). The study was approved by the Research Ethics Committee of Charité Universitätsmedizin Berlin (EA2/079/14) and informed consent was obtained from all parents/guardians. Bodyweight was measured with a digital scale (Soehnle, Nassau, Germany). Height was measured using a wall-mounted stadiometer (Keller, Leipzig, Germany). BMI was calculated (weight in kilograms divided by the square of the height in meters). Obesity was defined as BMI > 97th percentile according to German reference data. The degree of obesity was expressed as the standard deviation of the BMI (BMI-SDS). The general medical examination was performed and arterial blood pressure was measured in a supine position after 5 min rest using a Dinamap model V100 (GE Healthcare, Illinois, Chicago, USA). Results were interpreted using age-specific and sex-specific percentiles. Pubertal status was assessed according to Tanner’s criteria.

### Biochemical analyses and calculations

Blood sampling was performed after overnight fasting between 8 and 10 am. Fasting glucose, insulin level, uric acid, lipid state (total cholesterol; low-density lipoprotein (LDL); high-density lipoprotein (HDL); triglycerides), and kidney function parameters were measured by commercially available test kits in a certified laboratory (Labor Berlin Charité Vivantes GmbH, Berlin, Germany).

Insulin resistance was estimated by using the homeostasis model assessment of insulin resistance (HOMA-IR), according to Matthews et al. [25]: insulin (mIU/L) x glucose (mg/dl)/405. HOMA-IR was shown to correlate well with insulin resistance using the euglycemic-hyperinsulinemic clamp technique in obese as well as non-obese children and adults [26]. Insulin resistance was defined as sex- and age-adjusted HOMA-IR > 95th percentile, according to pediatric standard values by Allard et al. [27]. Metabolic syndrome was defined as proposed by the world health organization (WHO) for childhood [28]: obesity (BMI > 97th percentile) + (insulin resistance or impaired fasting glucose or impaired glucose tolerance) + (triglycerides > 150 mg/dl and/or HDL cholesterol <35 mg/dl and/or systolic and/or diastolic blood pressure ≥95th percentile). Hyperuricemia was defined as >5.9 mg/dl for female and >7.0 mg/dl for male adolescents.

### Melatonin measurement

To estimate the cumulative overnight melatonin secretion, 6-sulfatoxymelatonin (MT6s), the major metabolite of melatonin was measured in the first-morning urine and normalized to urinary creatinine levels to account for the urinary concentration. Several studies demonstrated that MT6s to creatinine ratio (MT6s:Cr) ratio correlates well with the cumulative nocturnal melatonin secretion [15, 16, 29]. Therefore, subjects were asked to bring a sample from the first-morning urine (between 06.30 and 07.00 am) on the day of examination, which was a regular school day. Samples were aliquoted, frozen, and stored at −80 °C. MT6s levels were measured in duplicates in the laboratory of pediatric endocrinology of Charité Universitätsmedizin Berlin using an enzyme-linked immunosorbent assay (ELISA) (BÜHLMANN laboratories AG, Schönenbuch, Switzerland). Urinary creatinine was measured in a certified laboratory (Labor Berlin Charité Vivantes GmbH, Berlin, Germany).

### Chronobiology

The chronobiological parameters were determined using the *Munich ChronoType Questionnaire* (MCTQ), which is the central instrument of an internet-based investigation of sleep behavior, chronotype, and social timing (https://www.thewep.org/documentations/mctq) and was shown to be a valid predictor of chronotype [30, 31]. For chronotype determination, the midpoint of sleep on workdays (MSW) and the midpoint of sleep on free days (MSF) were calculated [11]. Chronotype (MSFsc) was defined as MSF corrected for sleep deficit acquired during workdays. The discrepancy between biological and social timing, the social jetlag (SJL) was defined as the time difference between midsleep on free days (MSF) and midsleep on working days (MSW) [11]. Weekly average sleep duration was calculated as sleep duration during the week and during the weekend considering the number of free and working days ((SDw × WD + SDf × (7 − WD))/7).

Regular physical activity other than school sport was reported as well as daily electronic media consumption (television, computer, tablet or smartphone). Duration and daytime of overall media consumption including computer use for school were assessed by the questionnaire.

### Statistical methods

Statistical analyses were performed using SPSS (SPSS Inc., Chicago, Illinois, USA, version 27.0). Data are presented as mean ± standard deviation (SD) or median with 1st and 3rd quartile, depending on the distribution of continuous variables. Frequency is given in percentage (%) for categorical variables. Normality was tested by Kolmogorow–Smirnow test. Differences in medians were tested using nonparametric tests (Mann–Whitney U test for two independent variables, Kruskal–Wallis for more than two independent variables). To explore differences in multiple groups, post-hoc tests were performed (Bonferroni correction in case of variance homogeneity and Games–Howell post-hoc test in case of variance inhomogeneity).

To analyze the influence of the independent variables sex, age, and pubertal status, multiple linear regression analysis (enter model) was performed. The square root of MT6s:Cr ratio and the logarithm of triglyceride levels was applied in the regression models and correlations to ensure normal distribution. All analyses were explorative and p-values are interpreted as such.

## Results

### Baseline characteristics

The mean age of participants (*n* = 149) was 14.5 ± 2.1 years while 48% were boys. Insulin resistance was observed in 101 subjects (69%). In addition, metabolic syndrome according to the WHO criteria was identified in 53% of all subjects. Further baseline characteristics stratified to patients with and without insulin resistance are outlined in Table 1.

**Table 1:** Patients’ characteristics.

	All (*n* = 149)	Without IR (*n* = 46)	With IR^d^ (*n* = 101)	*p*-value
*Clinical characteristics*				
Sex (*n*; %)				
Male	71 (48%)	21 (46%)	48 (48%)	0.833^e^
Female	78 (52%)	25 (54%)	53 (52%)	
Age (years)^b^	14.7(12.8–16.3)	14.5 (12.1–16.1)	15.1 (13.3–16.3)	0.415^e^
Pubertal stage (*n*; %)^c^				
Prepubertal (I)	13 (10%)	6 (15%)	6 (6%)	0.293^e^
Pubertal (II,III)	27 (20%)	8 (20%)	19 (21%)	
Postpubertal (IV,V)	94 (70%)	26 (65%)	67 (73%)	
BMI (kg/m²)^b^	31.9 (29.5–37.2)	29.2 (27.7–32.0)	34.6 (30.7–38.9)	**<0.001**
BMI-SDS^a^	2.7 ± 0.6	2.4 ± 0.5	2.9 ± 0.5	**<0.001**
Systolic RR (mmHg)^b^	132 (122–141)	129 (120–138)	133 (126–142)	0.094
Diastolic RR (mmHg)^b^	68 (63–73)	68 (62–72)	68 (64–73)	0.684
*Chemical analysis*				
Triglycerides (mg/dl)^b^	93 (69–129)	69 (58–96)	102 (77–144)	**<0.001**
Total cholesterol (mg/dl)^b^	162 (147–184)	160 (138–179)	163 (152–186)	0.087
HDL cholesterol (mg/dl)^b^	48 (40–55)	53 (43–61)	46 (39–53)	**0.001**
LDL cholesterol (mg/dl)^b^	98 (85–117)	93 (73–111)	99 (86–119)	**0.044**
Uric Acid (mg/dl)^b^	5.5 (4.8–6.7)	5.2 (4.2–6.7)	5.7 (4.9–6.7)	0.050
MT6s:Cr ratio^b^	27.4 (17.2–37.4)	32.6 (23.0–44.0)	26.4 (15.8–35.0)	**0.006**

Bold values identify statistical significance *p* < 0.05.

*BMI* body mass index, *BMI-SDS* BMI standard deviation score, *HDL* high-density lipoprotein, *IR* insulin resistance, *LDL* low-density lipoprotein, *RR* Riva-Rocci blood pressure, *MT6s* 6-sulfatoxymelatonin, *Cr* creatinine.

^a^Data shown as mean ± SD.

^b^Data shown as median (25.–75.percentile).

^c^Pubertal stages (Tanner), *n* = 15 missing.

^d^defined as HOMA-IR > 95th percentile: according to Allard et al. [40]; *n* = 2 missing.

^e^χ^2^-Test.

The majority of patients (55%) reported no further weekly physical activity in addition to physical education at school. 33% declared to use electronic media less than 3 h per day, 48% between 3 and 6 h and media consumption of more than 6 h per day was identified in 19% of all subjects. Leisure time in front of a screen after 10 pm was seen in 46%.

### Overnight melatonin secretion, patient characteristics, and metabolic parameters

**Fig. 1:**
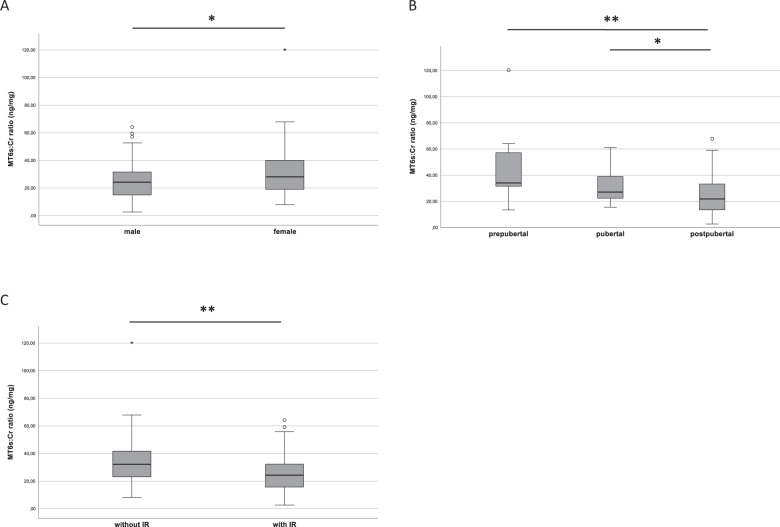
Melatonin secretion in regards to patients’ characteristics. **A** Shows differences of melatonin secretion in boys and girls. **B** Shows melatonin secretion in regards to different pubertal stages. Comparison of nocturnal melatonin secretion in patients without and with insulin resistance (**C**). Data are presented as boxplots. For statistical analysis Mann–Whitney U Test (**A, C**) and Kruskal–Wallis-Test (**B**) with Bonferroni correction was performed *p < 0.05; **p < 0.01.

Overnight melatonin secretion defined as MT6s:Cr ratio was significantly higher in girls (28.0 ng/mg; 1st quartile: 19.0 ng/mg; 3rd quartile: 40.2 ng/mg) compared to boys (24.3 ng/mg; 1st quartile: 15.0 ng/mg; 3rd quartile: 31.6 ng/mg; *p* = 0.010) (Fig. 1A). Older age was correlated with significantly lower MT6s:Cr ratio (Spearman’s r: −450; *p* < 0.001) while prepubertal children showed higher melatonin secretion compared to postpubertal adolescents: 34.1 ng/mg (1st quartile: 27.1 ng/mg; 3rd quartile: 58.3 ng/mg versus 21.8 ng/mg (1st quartile: 13.4 ng/mg; 3rd quartile: 33.3 ng/mg; *p* = 0.005) (Fig. 1B). Increased melatonin concentration was also observed in pubertal subjects (27.1 ng/mg; 1st quartile: 20.2 ng/mg; 3rd quartile: 41.7 ng/mg) compared to postpubertal adolescents (21.8 ng/mg; *p* = 0.045) while no significant differences were seen between prepubertal and pubertal subjects (34.1 ng/mg vs. 27.1 ng/mg; *p* = 0.699).

After adjustment for age, sex, and pubertal status, there was no significant association between MT6s:Cr ratio and BMI-SDS (*p* = 0.065).

Nocturnal melatonin secretion was significantly decreased in patients with insulin resistance (Fig. 1C). The median MT6s:Cr ratio was 24.3 ng/mg (1st quartile: 15.7 ng/mg; 3rd quartile: 33.0 ng/mg) among subjects with insulin resistance vs. 32.2 ng/mg (1st quartile: 22.7 ng/mg; 3rd quartile: 41.9 ng/mg) among those without insulin resistance (*p* = 0.006). After adjustment for age, sex, pubertal status this effect remained significant (*p* = 0.008).

Nocturnal melatonin secretion negatively correlated with uric acid levels (Pearson’s r: −0.247; *p* = 0.003), but not with lipid levels (triglycerides, total cholesterol; LDL; HDL). Performing a regression analysis, we identified that elevated uric acid levels (*p* = 0.029) and triglyceride levels (*p* = 0.012) showed associations with melatonin secretion (Table 2) after adjustment for sex, age, and Tanner stages. However, the presence of a metabolic syndrome did not seem to influence the melatonin secretion (*p* = 0.100; Table 2).

**Table 2:** Association of media consumption with chronobiology (MSFst and social jetlag) and melatonin secretion.

	B coefficient	*p*-value
*Chronotype (MSFst)*		
Media consumption after 10 pm	0.993	**0.001**
Duration of media consumption	1.070	**0.002**
3–6 h vs. <3 h	0.789	**0.004**
>6 h vs. <3 h	1.138	**0.001**
*Social jetlag*		
Media consumption after 10 pm	0.842	**0.004**
Duration of media consumption	1.057	**0.002**
3–6 h vs. <3 h	0.4034	0.131
>6 h vs. <3 h	1.072	**0.002**
*Melatonin secretion*		
Media consumption after 10 pm		
Duration of media consumption:	0.122	0.652
3–6 h vs. <3 h	−0.324	0.210
>6 h vs. <3 h	−0.486	0.145

Bold values identify statistical significance *p* < 0.05.

Regression analysis: Sex, age, and pubertal status were introduced as cofactors; *n* = 149. Linear regression model. B = unstandardized regression coefficient.

### Chronotype, metabolism, and media consumption

Significant negative correlations between nocturnal melatonin secretion and chronotype (Pearson’s r: −0.260; *p* = 0.002) as well as between melatonin secretion and social jetlag were observed (Pearson’s r: −0.248; *p* = 0.003).

**Fig. 2:**
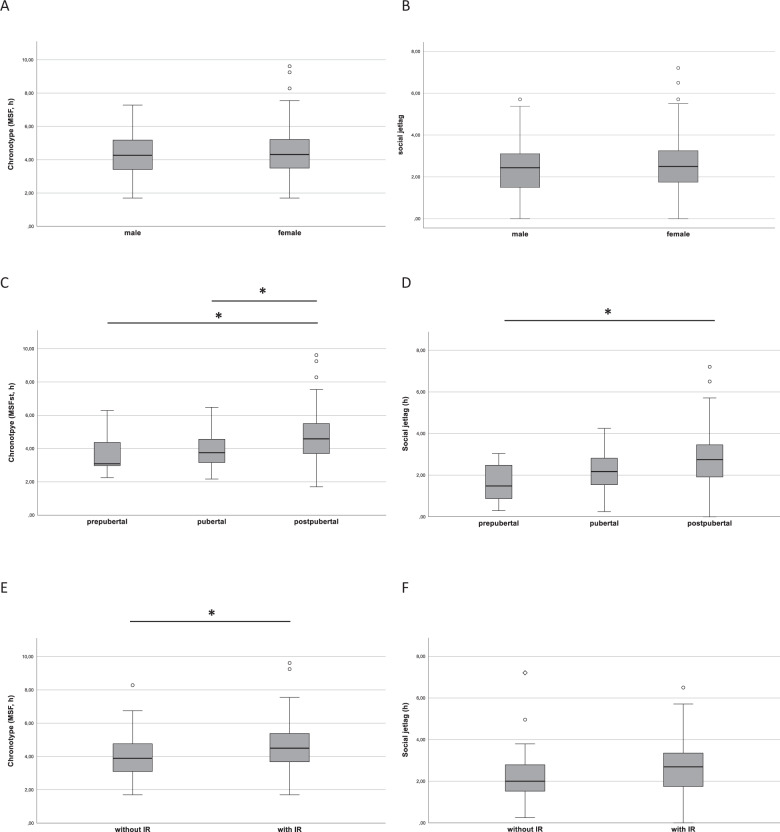
Comparison of chronobiologic parameters in males and females. **A** Differences of chronotype in males and females. **B** Differences of social jetlag in males and females. Data are presented as boxplots. For statistical analysis Mann–Whitney U Test was performed. Comparison of chronobiologic parameters stratified to pubertal stages. **C** Differences of chronotype. **D** Differences of social jetlag. Comparison of chronotype (**E**) and social jetlag (**F**) in patients with and without insulin resistance. Data are presented as boxplots. For statistical analysis Mann–Whitney U Test (**A**, **B**) and Kruskal–Wallis Test (**C**–**F**) was performed. Post-hoc tests are Bonferroni corrected. *p < 0.05.

Furthermore, increased age correlated with delayed chronotype (Spearman’s r: 0.369; *p* < 0.01) and increased social jetlag (Spearman’s r: 0.292; *p* < 0.01), while there were no differences between boys and girls regarding both chronotype and social jetlag. (Fig. 2A and B).

Patients with higher Tanner stages showed delayed chronotypes (*p* = 0.002; Fig. 2C). Differences were observed between prepubertal (3.0 h; 1st quartile: 2.7 h; 3rd quartile: 4.6 h) and postpubertal patients (4.5 h; 1st quartile: 3.6 h; 3rd quartile: 5.5 h; *p* = 0.014) as well as between pubertal (3.7 h; 1st quartile: 3.0 h; 3rd quartile: 4.6 h) and postpubertal patients (*p* = 0.023), while no differences were observed between prepubertal and pubertal patients (*p* = 0.999). Furthermore, patients with higher Tanner stages had increased social jetlag (*p* = 0.003) (see Fig. 2D). Differences were especially observed between prepubertal (1.5 h; 1st quartile: 0.8 h; 3rd quartile: 2.5 h) and postpubertal patients (2.7 h; 1st quartile: 1.9 h; 3rd quartile: 3.5 h) (*p* = 0.011) while differences between pubertal (2.2 h; 1st quartile: 1.5 h; 3rd quartile: 3.6 h) and postpubertal (*p* = 0.094) as well as between prepubertal and pubertal (*p* = 0.674) did not reach significance after Bonferroni correction.

The association between chronotype and insulin resistance is shown in Fig. 2E. After adjustment for sex, age and Tanner stages median MSF was 4.5 h (1st quartile: 3.6 h; 3rd quartile: 5.4 h) among subjects with IR versus 3.9 h (1st quartile: 3.1 h; 3rd quartile: 4.8 h) among those with unimpaired insulin secretion (*p* = 0.018). Therefore, patients with insulin resistance had a delayed chronotype for 38 minutes. In addition, social jetlag was increased in patients with insulin resistance (2.7 h; 1st quartile: 1.7 h; 3rd quartile: 3.3 h) compared to patients without insulin resistance (2.0 h; 1st quartile: 1.5 h; 3rd quartile: 2.8 h; Fig. 2F). However, this effect did not remain significant after adjusting for sex, age, and Tanner stages (*p* = 0.097).

Adjusted for sex, age and pubertal status no significant differences were observed between chronotype and BMI-SDS (*p* = 0.085), melatonin secretion (*p* = 0.212) or metabolic syndrome (*p* = 0.729).

In the next step, we examined a potential association between individual chronotype and media consumption. We identified significant associations of media consumption in the late evening (after 10 pm) and the duration of media consumption with chronotype and social jetlag (see Table 2). However, melatonin secretion showed no association with the daytime or duration of media consumption.

## Discussion

Several studies described a close relationship between melatonin secretion, obesity, and metabolic disorders in adults [13, 32, 33]. The purpose of this study was to assess the relationship between nocturnal melatonin concentration, chronotype, and metabolic parameters in obese adolescents with and without insulin resistance.

We identified that melatonin was negatively correlated with higher age and Tanner stages and that girls showed higher melatonin secretion compared to boys, which is in line with previous studies [34, 35]. Overall, our cohort showed a large inter-individual range in nocturnal melatonin secretion, which might be due to its complex secretion and interfering factors like season, chronotype, and the use of artificial light [15, 16, 36].

Here, obese patients with insulin resistance had lower nocturnal melatonin secretion. This effect remained significant after adjusting for pubertal status and is therefore most likely no reflection of an increase of insulin resistance during puberty as described by Moran et al. [37]. These results are further consistent with experimental and clinical data in adults that showed a strong association of melatonin and impaired insulin/glucose metabolism [13, 15, 16, 33, 38]. One of the underlying mechanisms could be polymorphisms of the melatonin receptor (*MTNR1B*) that are associated with a higher risk of glucose intolerance in children and adolescents [39]. Melatonin receptors (MT1 and MT2) are expressed in pancreatic β-cells where an inhibitory effect of melatonin on insulin secretion via cAMP and cGMP pathways has been described [33, 40–42]. Furthermore, melatonin also seems to have a direct impact on the synthesis, action, and secretion of insulin via regulating the expression and triggering of GLUT4 receptors [13]. A study in adolescent girls showed an association of later melatonin offset, insulin resistance, and polycystic ovarian syndrome [21] while no correlation was found between urinary MT6s and HOMA-IR in a group of South-Korean girls (6.3–12.4 years). However, this might be due to the age range and the fact that only a small number of patients showed relevant metabolic disorders [22].

Regression analysis further identified a significant association of triglyceride levels and melatonin secretion which was in line with several studies that described a hypolipidemic effect of melatonin in adult patients with diabetes mellitus type 2 [17, 18, 43]. Furthermore, patients with elevated uric acid levels showed lower melatonin secretion, which is also in accordance with adult studies [15]. As elevated uric acid levels are also known to be an independent risk factor for cardiovascular diseases [44], alterations of melatonin secretion may represent a possible link: a study by Mayo et al. indicated that melatonin is able to lower systemic inflammation caused by elevated uric acids levels via inhibition of cyclooxygenase 2, which may reduce mitochondrial dysfunction [45]. Interestingly, we only found relevant associations of individual components of the metabolic syndrome (insulin resistance, hypertriglyceridemia, and hyperuricemia) but not with the complete metabolic syndrome, which is in contrast to several studies in adults [17, 46]. Moreover, no significant correlation between obesity and melatonin secretion was observed, possibly due to the small range of BMI-SDS. However, strong evidence from experimental data exists that melatonin is involved in energy metabolism and body fat regulation [47, 48]. For instance, in experimental studies in rats, pinealectomies led to increased bodyweight, impaired glucose tolerance, and elevated insulin levels [49], whereas melatonin substitution in diabetes-prone rats prevented the development of diabetes and was even able to reduce bodyweight [47]. However, whether melatonin concentrations are affecting bodyweight and development of obesity directly and by which mechanism is discussed controversially [22, 35].

In several studies in adults, treatment with melatonin was able to show a significant reduction of bodyweight and oxidative stress as well as an improvement of lipid profile, insulin sensitivity, and hepatic parameters [17, 50]. Most of these studies used melatonin as an adjuvant, suggesting that it is most effective when combined with a multimodal lifestyle therapy [50]. However, other studies were not able to reproduce the beneficial effects of melatonin supplementation or questioned the longevity of these effects. It is also not clear if all patients or only those with the particularly low secretion of melatonin could benefit from a supplementation [50].

Our results further demonstrated that higher age and Tanner stages were associated with later chronotype and increased social jetlag, which was also observed in previous studies in this age cohort [7, 8]. While we did not observe any sex-related differences, we identified a significant correlation between delayed chronotype and insulin resistance. These results support studies in adults, which provided evidence that sleep disturbances are associated with abnormal glucose metabolism [51]. A large Finnish study (25–74 years) detected that subjects with late chronotypes had a 2.5-fold increased risk for diabetes mellitus type 2 compared to individuals with an earlier chronotype [52]. Additionally, Reutrakul et al. identified that late chronotype is associated with poorer glycemic control in patients with diabetes mellitus type 2 independent of sleep quality or duration [53]. These results suggest that the biological clock is playing a role in metabolic regulation [53]. Studies conducted in shift workers, provide additional evidence for a link between circadian misalignment and metabolic disorders as they found that shift work is associated with a significantly higher risk of developing diabetes mellitus type 2 and other metabolic diseases [54]. One potential molecular link between sleep disturbance and metabolic diseases is melatonin as we observed an association between overnight melatonin secretion and the presence of insulin resistance. We further identified negative correlations between nocturnal melatonin secretion and individual chronotype as well as social jetlag. However, other hormones (e.g., cortisol, glucagon, leptin, ghrelin, adiponectin, growth hormone) that also underlie a circadian rhythm [32] may additionally play a role in that: for example, studies showed that chronic sleep loss leads to an increase of ghrelin and a decrease of leptin possibly resulting in an increase of appetite and a decrease of satiety [55].

One common underlying cause of induced altered sleeping behavior and low melatonin secretion is the use of artificial light. In our patient cohort, late chronotype and social jetlag were associated with higher and later (after 10 pm) media consumption, which was consistent with previous studies [19]. This delay of the individual circadian rhythm may result in sleep disorders like insomnia or daytime sleepiness with related physical and psychological disorders [20]. Furthermore, worse academic performance [56] and higher incidences of traffic accidents [57] have been observed in this age group which is why the American Academy of Pediatrics even suggested postponing the daily start of school for adolescents [58].

### Limitations and conclusion

In the present study, we identified an association between melatonin secretion and insulin resistance, but the data do not allow any conclusion about the direction. However, genetic studies examining melatonin receptor alterations rather suggest an influence of melatonin on insulin resistance and ß-cell function than vice versa [59].

Furthermore, assessing melatonin secretion by measuring its main metabolite MT6s in morning urine samples and normalizing it to urine creatinine levels does not allow any information on timing or amplitudes of secretion. However, after many years of experience with 24 h urine samples in a pediatric outpatient setting, we believe that the applied method is the most robust and accurate available method for this group of adolescent patients as is it easy to use and non-invasive. The small range of BMI-SDS and the lack of a normal weight control group may have affected the power to detect associations between nocturnal melatonin secretion and obesity as well as between chronotype and obesity. In addition, conclusions regarding different Tanner stages are limited since prepubertal and pubertal subjects were underrepresented in our patient cohort. Furthermore, we did not take seasonality into account. Although there is evidence that MT6s output is not affected by season [36], a bias due to different lengths of days during data collection cannot be fully excluded.

In conclusion, this is the first study that identified associations of lower nocturnal melatonin secretion, late chronotype, and insulin resistance in adolescents with obesity. These findings support the growing evidence for a substantial interaction of melatonin secretion and chronotype with metabolism. Interestingly, we did not observe a correlation between BMI-SDS and nocturnal melatonin levels. This might argue for a bodyweight independent impact of melatonin on metabolic function, specifically in regard to the risk of developing insulin resistance. Based on the knowledge about the relationship between melatonin secretion, chronotype, and social jetlag, it might be of importance to integrate strategies to reduce social jetlag by adapting sleeping behavior with the individual chronotype into multimodal lifestyle therapy of adolescents with obesity. This implies changes in media consumption time especially in the evening, exposure to daylight, and alignment of environmental factors like daily school start time with the age-dependent chronotype.
